# Impact of tiered measures on social contact and mixing patterns of in Italy during the second wave of COVID-19

**DOI:** 10.1186/s12889-023-15846-x

**Published:** 2023-05-19

**Authors:** Michele Tizzani, Alessandro De Gaetano, Christopher I. Jarvis, Amy Gimma, Kerry Wong, W John Edmunds, Philippe Beutels, Niel Hens, Pietro Coletti, Daniela Paolotti

**Affiliations:** 1 ISI Foundation, Turin, Italy; 2 London School of Hygiene and Tropical Medicine, London, UK; 3 Centre for Health Economic Research and Modeling Infectious Diseases (CHERMID), Vaccine and Infectious Disease Institute, University of Antwerp, Antwerp, Belgium; 4 UHasselt, Data Science Institute and I-BioStat, Hasselt, Belgium

**Keywords:** COVID-19, NPI, Non-pharmaceutical interventions, Social contact patterns, Contact matrix, Governmental response, Reproduction number, Contact survey, Italy

## Abstract

**Background:**

Most countries around the world enforced non-pharmaceutical interventions against COVID-19. Italy was one of the first countries to be affected by the pandemic, imposing a hard lockdown, in the first epidemic wave. During the second wave, the country implemented progressively restrictive tiers at the regional level according to weekly epidemiological risk assessments. This paper quantifies the impact of these restrictions on contacts and on the reproduction number.

**Methods:**

Representative (with respect to age, sex, and region of residence) longitudinal surveys of the Italian population were undertaken during the second epidemic wave. Epidemiologically relevant contact patterns were measured and compared with pre-pandemic levels and according to the level of interventions experienced by the participants. Contact matrices were used to quantify the reduction in the number of contacts by age group and contact setting. The reproduction number was estimated to evaluate the impact of restrictions on the spread of COVID-19.

**Results:**

The comparison with the pre-pandemic baseline shows a significant decrease in the number of contacts, independently from the age group or contact settings. This decrease in the number of contacts significantly depends on the strictness of the non-pharmaceutical interventions. For all levels of strictness considered, the reduction in social mixing results in a reproduction number smaller than one. In particular, the impact of the restriction on the number of contacts decreases with the severity of the interventions.

**Conclusions:**

The progressive restriction tiers implemented in Italy reduced the reproduction number, with stricter interventions associated with higher reductions. Readily collected contact data can inform the implementation of mitigation measures at the national level in epidemic emergencies to come.

**Supplementary Information:**

The online version contains supplementary material available at 10.1186/s12889-023-15846-x.

## Introduction

The COVID-19 pandemic caused by the new coronavirus SARS-COV-2 has created a global crisis that has caused millions of deaths around the world and has imposed an unprecedented burden on the healthcare and economic infrastructure of most countries. During the first phases of the pandemic, due to the lack of specific and effective pharmaceutical treatments or vaccines, governments, and public health institutions relied on interventions aimed at reducing individual and collective social contacts among the general population, in parallel with the enforcement of hygiene measures, such as wearing a mask [1]. How effective were these measures at reducing epidemiologically relevant contact patterns? What is the relationship between the stringency of restrictions and the number of contacts made?

In this context, the CoMix initiative was an unprecedented Europe-wide [2] effort consisting of multi-country social contact surveys in representative panels of individuals in terms of age, gender, region of residence, and for most countries either socio-economic status, occupation, or educational attainment. This work presents the results of a longitudinal survey of the Italian population in the scope of the CoMix initiative evaluating the impact on social contacts of physical distancing measures. As far as restriction measures are concerned, the case of Italy is especially interesting as the public health system is fragmented in regional governments which are ultimately responsible for public-health-related decisions. During the COVID-19 crisis, this led to the creation of a tiered regional system for the implementation of restrictive measures that corresponded to the introduction of color-coded “zones”. organized in progressively restrictive tiers (color-coded as yellow, orange, and red) imposed by the Italian government. The measures were implemented on a regional basis according to real-time epidemiological risk assessments [3]. By measuring epidemiologically relevant contact patterns (proxied by the number of face-to-face conversations and physical contacts) in the different regions over time this study aims to quantify the impact of these tiered restrictions on contacts and the COVID-19 reproduction number.

## Methods

### Survey methodology

Digital surveys were administrated to a panel of participants (representative of the general population) aimed at determining changes in contact patterns in everyday life settings (e.g. home, workplace, school, etc.). The surveys were conducted by the market research company Ipsos. Participants were recruited through email invitations to adult members of Ipsos online panels who could fill the survey for themselves or on behalf of children (i.e. an individual aged less than 18) in their household. The surveys were submitted by participants every two weeks between the end of December 2020 and April 2021, for a total of seven waves of data collection. The sample was representative of the Italian population by age, sex, geographical location, and socioeconomic status (deviations of $5\%$ were allowed in each stratum). Furthermore, records of participants’ occupations, health status, and attitudes toward non-pharmaceutical intervention measures and restrictions were also collected. Participants were also asked to specify whether they, their household members or their contacts had tested positive for COVID-19 at any point during the data collection and if this had led to self-isolation/quarantine. Participants were asked to record all contacts they had during the 24 hours before each survey. Participants were asked to provide information about the age, gender, physical contact, duration, and frequency of each contact as well as if physical contact occurred. Participants were also asked to record contact locations (home, work, school, leisure activities, other places) and specify whether the contact occurred outdoors or indoors. To allow for reporting a high number of contacts, participants were also asked to report contacts with a group. These group contacts were restricted to three possible locations (work, school, others), to three age categories (0-18, 19-65, and 65+ years), and to be physical or not.

### Stratification by color-code

Participants were stratified by color code, i.e. according to the color assigned to their region of residence during the reporting period. Table 1 shows the criteria for the definition of the color codes and the corresponding interventions [3]. The color of each region during each data collection wave is shown in SI (Fig. 6). We excluded the white zone from the analysis due to a lack of data.

**Table 1:** NPIs conditions. Conditions and allowed activities for each zone

		Yellow Zone	Orange Zone	Red Zone
**Conditions**	Weekly incidence (W.I.) every 100000 cases	50 < W.I. $\le$ 150	150 < W.I. $\le$ 250	W.I. > 250
		or	and	and
	Occupation rate of beds in hospitals (rate for ICU)	$\le$ 30% (ICU $\le$ 20%)	$\le$ 40% (ICU $\le$ 30%)	$\ge$ 40% (ICU $\ge$ 30%)
**Movements and public transport**				
Travel between 22.00 and 5.00		✗	✗	✗
Travel between municipalities and regions		✓	✗	✗
Travel within the municipality		✓	✓	✗
Public transport reduced to 50%		✓	✓	✓
**Activities and Restaurants**				
Access to shopping centers during working days		✓	✗	✗
Access to museums, gyms, theaters and cinemas		✗	✗	✗
Access to sport centers		✓	✓	✗
Access to bars and restaurants before 18.00		✓	✗	✗
Takeaway until 22 and home delivery		✓	✓	✓
**Education** ^a^				
On-site learning for high schools (Age: 14+)		✗	✗	✗
On-site learning for middle schools (Age: 11-14)		✓	✓	✗
On-site learning for primary schools (Age: 0-11)		✓	✓	✓

^a^ In some cases, restrictions could vary slightly from region to region

### Contact Matrices

Based on the information reported in the surveys, age-stratified contact matrices were calculated. Contact matrix elements were computed according to the following equation:

$$\begin{aligned} m_{ij} = \frac{\sum ^{T_i}_{t=1}{w_{it}y_{ijt}}}{\sum ^{T_i}_{t=1}{w_{it}}} \end{aligned}$$

where $y_{ijt}$ denotes the reported number of contacts experienced by participant *t* of age *i* with someone of age *j*. $T_i$ denotes all participants of age *i*, and $w_{it}$ is the post-stratification weight. This accounts for possible under- and over-sampling of age categories or survey day type (either weekday or weekend). When computing the contact matrix for a specific wave/zone we included only participants reporting contacts in that wave/zone and their corresponding contacts. We used the R library *Socrates* [2, 4], based on the *socialmixr* package, that includes population information from the Nation’s World Population Prospect [5]. To account for sampling variability, we applied a bootstrapping procedure to the sample with $n=10000$. When not available, the information about the age of the contacts was sampled out of the age distribution of the reported individual contacts. Missing contact ages were sampled from the reported age groups. The pre-pandemic baseline was defined by the POLYMOD dataset for Italy [6]. Contact settings were defined using the following possibilities: (i) home, i.e., whether the contact occurred inside a house, not necessarily corresponding to the participant’s household, (ii) workplace contacts, and (iii) other, i.e., all contacts occurred in none of the previous locations. In the latter case, to compute the contact matrices, we considered only the contacts that the participants had in specific settings. In particular, the matrix element for a given setting *S* is given by:

$$\begin{aligned} m^S_{ij} = \frac{\sum ^{T_i}_{t=1}{w_{it}y^S_{ijt}}}{\sum ^{T_i}_{t=1}{w_{it}}} \end{aligned}$$

where $y^S_{ijt}$ is the number of contacts of the participant *t* of age *i* with someone of age *j* in the setting *S*. Finally, we averaged all the bootstrap iterations and considered the mean value for each matrix element.

### Reproduction number

$R_0$ can be estimated as the dominant eigenvalue of the next-generation matrix using the social contact hypothesis [7] which states that the next-generation matrix is proportional to the contact matrix. In this case, the number of infectious contacts is proportional to the number of social contacts through an age-dependent factor $\varvec{q}_i$, and thus, the next-generation matrix can be derived from the contact matrix. The pre-pandemic baseline for contacts was defined by the POLYMOD contact matrices for Italy. The relative reduction of $R_0$ can thus be determined as the relative reduction in the largest eigenvalue of the contact matrix given by

$$\begin{aligned} R_0 = R_{0_{ref}} R_{0_{ratio}} = R_{0_{ref}} \frac{\rho (q M)}{\rho (q M_{P})} \end{aligned}$$

where $R_{0_{ref}}$ is the value of $R_0$ before COVID-19 restrictions (we used 2.96 with sd 0.11 [8]), $\rho (q M)$ and $\rho (q M_{P})$ are the spectral radius of the next generation matrices of the CoMix data ( possibly restricted to a given wave or zone) and the POLYMOD data. Symmetric contact matrices were assumed. Any difference in the transmission of the virus among different age groups is assumed to be negligible, i.e. it was assumed that *q* was not age dependent, which means that $\rho (q M) = q \rho (M)$ and a simplified version of the formula above is obtained. However, an age sensitivity analysis using different susceptibility and infectiousness values taken from the literature [9, 10] was performed.

Finally, to assess the differences between different zones, we compared the distribution of $R_0$ calculated from the bootstrap sample ($n=10000$) of contact matrices for each zone.

## Results

### Sample composition

The total number of participants was 1617 who provided 7657 answers to the survey, giving data on 23003 contacts. Table 2 shows the distribution of the participants according to age, gender, and household size. The number of participants per wave started at 1599 during wave 1 and declined to 591 in wave 7. The two waves targeted at children recorded $\approx$ 300 responses. Good representativity of the Italian population in terms of age, sex, and geographical location (reported separately in Table 1 in SI ) is observed, with small wave-to-wave variations. The distribution of participants according to age groups, household sizes, and sex in the different zones is shown in Table 4 (a). The largest number of participants is observed when yellow-coded restrictions are in place (3141), with the number of participants when orange- and red-coded restrictions are in place amounting to 2228 and 1646, respectively. Participants in white-coded regions are 39 and are therefore excluded from the analysis. The average age of the participants was 49.3 (standard deviation $sd = 16.4$, $max=93$), with a median of 49 years, an interquartile range of $[36-64]$. The majority of the participants were male ($53.3\%$). Most of the participants did not live alone $(98.2 \%)$. The most common household arrangement was two adults without children $(24.6\%)$. The predominant employment statuses were employed full-time $(25.4\%)$ or retired $(27.8\%)$.

**Table 2:** Distribution of participants. Distribution of participants by wave, age group, gender, and household size

Wave	1	2	3	4	5	6	7	1C	2C
All	1559	1324	1125	955	811	689	591	306	297
Age Group									
0-4								84 (28 %)	77 (26 %)
5-17								222 (72 %)	211 (71 %)
18-29	240 (15 %)	184 (14 %)	151 (13 %)	150 (16 %)	107 (13 %)	145 (21 %)	84 (14 %)		
30-39	206 (13 %)	170 (13 %)	140 (12 %)	140 (15 %)	100 (12 %)	137 (20 %)	61 (10 %)		
40-49	323 (21 %)	266 (20 %)	238 (21 %)	243 (25 %)	173 (21 %)	164 (24 %)	131 (22 %)		
50-59	239 (15 %)	206 (16 %)	178 (16 %)	190 (20 %)	143 (18 %)	64 (9 %)	87 (15 %)		
60+	551 (35 %)	498 (38 %)	418 (37 %)	232 (24 %)	288 (36 %)	179 (26 %)	228 (39 %)		
Gender									
F	745 (48 %)	615 (46 %)	523 (46 %)	438 (46 %)	384 (47 %)	310 (45 %)	272 (46 %)	302 (99.7%)	292 (99%)
M	810 (52 %)	709 (54 %)	601 (54 %)	517 (54 %)	426 (53 %)	379 (55 %)	319 (54 %)	1 (0.3%)	4 (1%)
HH size									
1	184 (12 %)	167 (13 %)	140 (12 %)	106 (11 %)	109 (13 %)	88 (13 %)	83 (14 %)		
2	512 (33 %)	446 (34 %)	385 (34 %)	281 (30 %)	287 (35 %)	225 (33 %)	193 (33 %)	13 (4 %)	8 (3 %)
3 - 5	774 (50 %)	641 (48 %)	546 (48 %)	512 (54 %)	381 (47 %)	343 (50 %)	285 (48 %)	254 (83 %)	250 (84 %)
5+	89 (6 %)	70 (5 %)	54 (5 %)	55 (6 %)	34 (4 %)	33 (5 %)	30 (5 %)	39 (13 %)	38 (13 %)

#### Contact patterns and the impact of restrictions

**Fig. 1:**
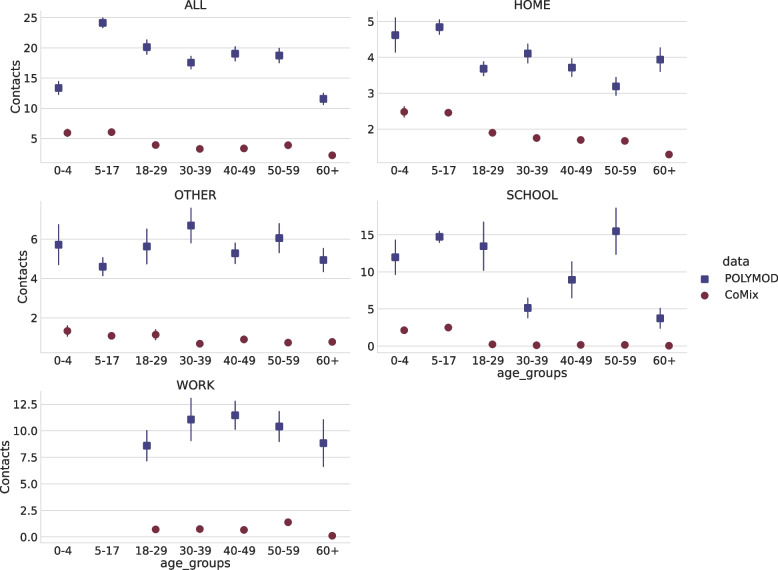
Average number of contacts. Average number of contacts from CoMix and POLYMOD data in all locations (ALL), at home (HOME), at other locations (OTHER), at school (SCHOOL), and at work (WORK). Point symbols

**Fig. 2:**
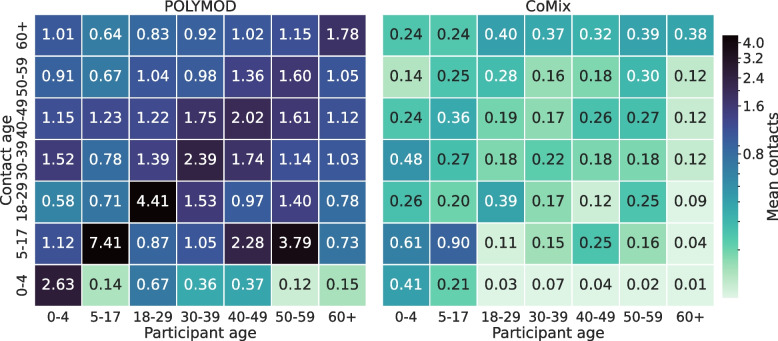
Contact matrices. Contact matrices for the adult population from the POLYMOD and CoMix data

**Fig. 3:**
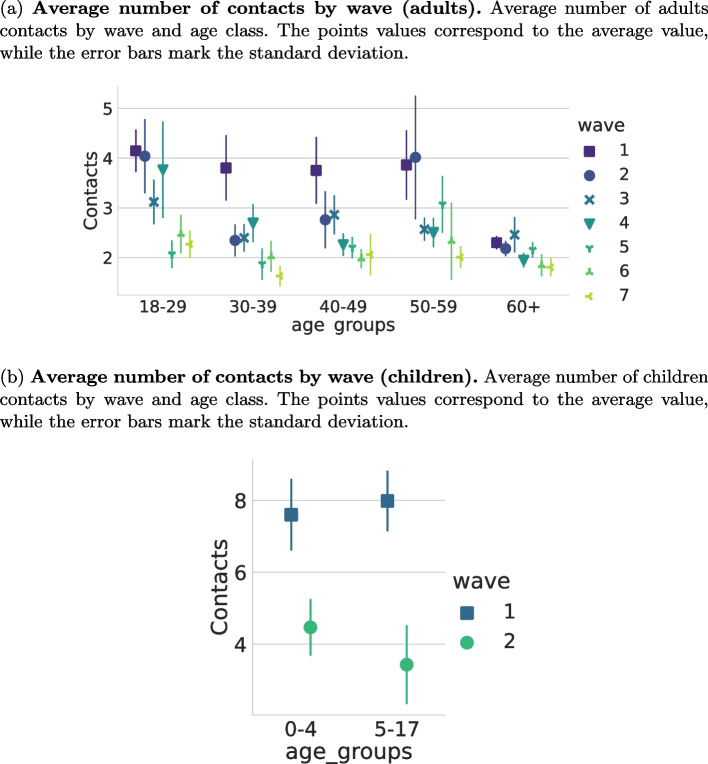
(a) Average number of contacts by wave (adults). Average number of adults contacts by wave and age class. The points values correspond to the average value, while the error bars mark the standard deviation. (b) Average number of contacts by wave (children). Average number of children contacts by wave and age class. The points values correspond to the average value, while the error bars mark the standard deviation

**Fig. 4:**
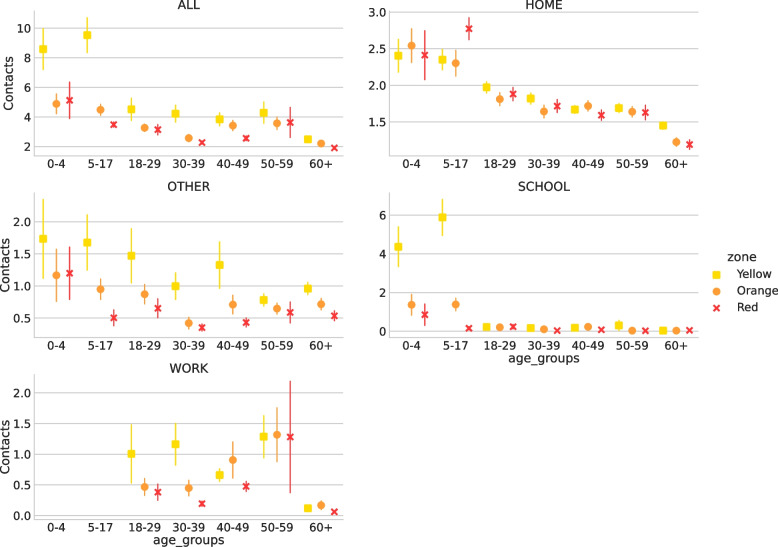
Average number of contacts by zones. Average number of contacts broken down by age and zone for contacts reported at all locations (ALL), at home (HOME), at other locations (OTHER), at school (SCHOOL), and at work (WORK). Point values correspond to the average value, while the error bars mark the sd. The color code indicates the NPIs, yellow for the lowest stringency regime, while red for the highest

**Fig. 5:**
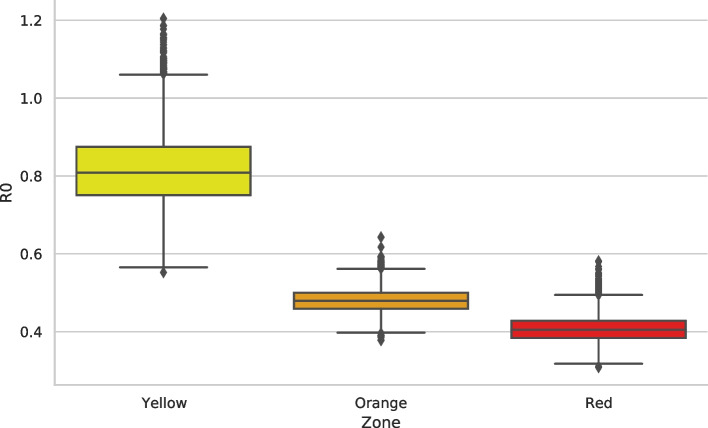
Reproduction number. Reproduction number computed for each zone. The color code indicates the NPIs, Yellow for the lowest stringency regime, and Red for the highest

Contacts were stratified by age group, sex, waves, and household size. The descriptive comparison between CoMix survey and POLYMOD with respect to these categories is shown in Table 3. Figure 1 shows the average number of contacts for CoMix and POLYMOD for each age group in several settings. The differences between the two data sets are quite evident in all the settings even though the gap is less strong for home settings. Figure 2 compares the overall age-stratified contact matrices obtained by CoMix with those obtained for POLYMOD. Notably, while during the pre-pandemic period, most contacts happened between the same age classes (thus confirming the assortative mixing of individuals [6]), this is not entirely true for the contact matrix in the pandemic time where off-diagonal values are more heterogeneous with respect to the pre-pandemic period. In particular, the average number of contacts with the elderly during the pandemic is homogeneously distributed between the age groups of the participants and is generally higher than with other classes. The contact matrices for different waves, zones, and settings can be found in Figs. 1, 2, and 3 of the SI respectively. Figure 3a shows how, for each age group, the average number of contacts changed over time. There was a decline in reported contacts in each age group over the period of study. Figure 4 shows how average reported contact rates vary according to restriction tires. Reported contact rates were higher in the yellow zone for both the orange and red zones, which had significantly stricter measures, as described in Table 4 (b). While more contacts were reported in the yellow zone consistently across all age groups, the difference shows an age-specific effect. The largest difference in the number of contacts is reported for children aged 0-17 between the yellow and orange zone and is determined by the large difference in contacts at school reported (Fig. 4). Contacts at home, do not show appreciable differences across the three different zones, with the exception of children aged 0-17 reporting more contacts in the red zone than in yellow or orange zones. Contacts at “other” locations present the largest difference between zones, the more so the younger the participant. Overall, a higher number of contacts was reported during the first wave of data collection for all age groups, except for the 60+ class, compared to the last wave of reporting. The average number of contacts was heterogeneous across the age groups for all waves. In particular, and regardless of the wave of data collection, elderly participants (60+) reported a lower number of contacts than other age classes, with a distribution showing less heterogeneity than the other age classes. The most relevant differences for this age group are for work contacts, with a markedly lower number than the other age classes, consistent with the fact that a considerable fraction of this group consists of retired people. Furthermore, the measured contact patterns are not symmetric with respect to age classes. In particular, elders were more being contacted by other participants than reaching out to them. This might be caused by the household structures consisting of elders living with their families. Finally, the impact of restrictions on the severity of the epidemic, measured by the change in the colored zones of the reproduction number $R_0$ is shown in Fig. 5. It can be seen that $R_0$ is significantly lower for the red and orange zones compared to the yellow zone, which had the least restrictive set of interventions. Statistical testing confirms that the difference is statistically significant (KS test, $p<0.0001$). Similarly, the difference between the orange and red zones is also significant (KS test, $p<0.0001$). The impact of the dependency in *q* is shown in SI (Fig. 4 SI ). Results consistently show a decrease in $R_0$ when computed for the yellow, orange, and red zones.

**Table 3:** Average number of contacts. The average number of contacts by wave for CoMix and Polymod

	CoMix (IQR)									Polymod (IQR)
Wave	1	2	3	4	5	6	7	1C	2C	1
All	3.98 (1-4)	3.16 (1-3)	3.06 (1-3)	3.14 (1-3)	2.62 (1-3)	2.54 (1-3)	2.29 (1-3)	8.4 (3-8)		18 (9-24)
Age Group										
0-4								7.75 (3-9)	4.21 (2-5)	15.05 (7-18)
5-17								8.08 (3-9)	3.76 (3-5)	24.86 (15-35)
18-29	4.69 (2-5)	3.39 (2-4)	3.64 (2-4)	5.21 (1-6)	2.56 (1-3)	3.18 (1-3)	2.65 (1-3)			20.91 (11-28)
30-39	4.9 (2-5)	2.98 (1-3)	3.03 (1-4)	3.33 (1-4)	2.48 (1-3)	2.69 (1-3)	2.3 (1-3)			18.39 (11-24)
40-49	4.39 (1-5)	4.11 (1-5)	3.87 (1-4)	3.12 (1-4)	2.93 (1-3)	2.65 (1-3)	2.89 (1-3)			19.26 (9-27)
50-59	5.52 (1-6)	4.01 (1-4)	3.19 (1-4)	3.11 (1-4)	3.76 (1-4)	3.62 (1-34	2.53 (1-3)			18.9 (9-25)
60+	2.77 (1-3)	2.62 (1-3)	2.68 (1-3)	2.22 (1-3)	2.39 (1-3)	2.03 (1-3)	2.12 (1-3)			14.63 (7-19)
Gender										
F	3.71 (1-4)	3.36 (1-4)	3.05 (1-4)	3.68 (1-4)	2.87 (1-4)	2.89 (1-4)	2.37 (1-3)	8.65 (3-9)	3.95 (2-4)	17.87 (9-24)
M	4.48 (1-5)	3.23 (1-4)	3.3 (1-4)	2.9 (1-4)	2.69 (1-4)	2.54 (1-4)	2.51 (1-4)		3.75 (2-4)	18.89 (10-25)
HH size										
1	3.53 (1-2)	1.97 (1-3)	1.92 (1-3)	2.07 (1-3)	1.61 (1-3)	1.35 (1-1)	1.64 (1-3)			15.02 (5-22)
2	2.98 (1-3)	2.54 (1-3)	2.12 (1-3)	3.39 (1-4)	2.63 (1-3)	2.3 (1-3)	1.61 (1-2)	5.17 (1-5)	1.5 (1-2)	16.87 (8-22)
3 - 5	4.82 (2-5)	4.04 (2-4)	3.97 (2-4)	3.26 (2-3)	3.14 (2-3)	3.14 (2-4)	3.05 (2-4)	8.85 (3-8)	3.81 (2-4)	19.04 (11-25)
5+	5.54 (4-6)	4.33 (3-5)	6.07 (4-7)	4.8 (4-6)	3.79 (2-5)	4.42 (3-6)	4.37 (3-5)	8.97 (4-9)	5.53 (4-6)	20.0 (11-27)

**Table 4:** Distribution of participants and contacts by zone, age group, gender, household size

Zone	Orange	Red	Yellow
(a) Total number of participants and percentage			
All participants			
	2228	1646	3141
Age group			
0-4	58 (2 %)	51 (3 %)	52 (2 %)
5-17	165 (7 %)	123 (7 %)	145 (4 %)
18-29	349 (16 %)	281 (17 %)	429 (14 %)
30-39	277 (12 %)	242 (15 %)	424 (14 %)
40-49	476 (21 %)	352 (21 %)	704 (22 %)
50-59	362 (16 %)	226 (14 %)	512 (16 %)
60+	764 (34 %)	545 (33 %)	1072 (34 %)
Gender			
F	1286(52 %)	956(52 %)	1622(49 %)
M	1169(48 %)	865(48 %)	1710(51 %)
HH size			
1	309 (14 %)	191 (12 %)	374 (12 %)
2	758 (31 % )	532 (29 % )	1047 (31 % )
3 - 5	1243 (51 % )	994 (55 % )	1729 (52 % )
5+	147 (6 % )	105 (6 % )	188 (6 % )
(b) Average number of contacts and IQR			
All contacts			
	2.93 (1-3)	2.58 (1-3)	3.56 (1-4)
Age group			
0-4	6.33 (2-7)	4.32 (2-4)	8.77 (3-9)
5-17	5.82 (3-6)	3.5 (2-4)	10.91 (3-12)
18-29	3.23 (1-4)	3.26 (1-3)	4.62 (2-4)
30-39	2.79 (1-3)	2.45 (1-3)	4.17 (1-4)
40-49	3.61 (1-4)	2.76 (1-3)	3.98 (1-4)
50-59	3.72 (1-4)	3.62 (1-3)	4.12 (1-4)
60+	2.49 (1-3)	2.19 (1-3)	2.68 (1-3)
Gender			
F	2.99 (1-3)	2.72 (1-3)	3.73 (1-4)
M	3.16 (1-3)	2.74 (1-3)	3.63 (1-4)
HH size			
1	1.61 (1-1)	1.35 (1-1)	3.04 (1-2)
2	2.32 (1-2)	2.11 (1-2)	3.02 (1-3)
3 - 5	3.86 (2-4)	3.19 (2-3)	4.14 (2-4)
5+	4.82 (4-5)	5.17 (3-5)	4.82 (4-6)

## Discussion

The pandemic strongly affected contact patterns among the population, as restrictions in most countries tried to reduce social mixing and mitigate the spreading of the COVID-19 epidemic. The CoMix initiative was aimed at quantifying this unprecedented change in social behavior with a Europe-wide effort based on contact surveys administered to representative panels in 19 European countries [2]. Results have shown how in countries such as the UK [11], Belgium [12] and the Netherlands [13], the CoMix initiative was capable of capturing the evolution of contact patterns across the various phases of an ever-changing pandemic. This was the case also for Italy, as detailed by the contact patterns across different age groups and in different settings across various sets of restrictions. To face the unfolding of the COVID-19 pandemic, the Italian government developed progressively restrictive tiers (coded as yellow, orange, and red) imposed on a regional basis according to real-time epidemiological risk assessments, based on several indicators [3]. The results show a significant impact in terms of the reduction of social mixing with stricter tiered measures. In particular, the differences were stronger between the yellow and the other two zones, and while more contacts were reported in the yellow zone independently of the age group, the difference between the red and orange zones is larger for children aged 5-17 and middle-aged population. In particular, the large reduction in contacts for children (i.e. 0-17) between yellow and orange/red zones is driven by the number of contacts at school. Although the policy of school closing is implemented at the regional level (no variation between the same color code is possible), our result shows an overall effect in school contacts depending on the color-code restrictions. In-house contacts, however, are similar across the different zones consistently with the fact that home visits were at least partially allowed even in the red zone. The only, and notable, exception here are the contacts at home of children aged 5-17, which increase from orange to red zone. This is consistent with the closure of middle schools, which leads children to stay at home under the supervision of an adult, hence increasing their contacts at home. Other studies in Europe [12–15] and worldwide [16, 17] have reported a similar reduction in the number of contacts during COVID-19 social distancing, in particular highlighting the reduction in non-household contacts. Specifically, the number of contacts measured in red zones is comparable to the 2-5 average contacts per day reported during an earlier phase of the pandemic, when stay-at-home mandates were in place [17]. The impact of restrictions on contact patterns and therefore on the spread of COVID-19 is also confirmed by the analysis of R0. Based on the social contact hypothesis, considering POLYMOD contact data as the baseline for pre-pandemic contacts and an R0 of 2.96 (2.73-3.17 95% CI) [8], we were able to link the reduction in contact patterns to a value of R0. We obtained average values of R0 that are lower than 1 for every color-code of interventions (Fig. 5), however with confidence interval including one for the yellow zone. Results from linear mixed model informed by mobility data [18] report comparable reductions in the reproduction number, confirming the efficacy of the tiered system. The additional insight that our study provides is the location and age-specific contribution to the reduction, with contacts of working adults at work and of children at school responsible for the large reduction in R0 between yellow and orange zones. In fact, more stringent intervention measures further reduce the reproduction number. Unfortunately, we did not collect enough data to estimate the reduction of R0 for white zones. Our results show that these restrictions, adopted during Winter 2020 and Spring 2021, have contributed to drastically reducing the reproduction number. The limitations of this work are discussed in the following. First, the least strict level of intervention was excluded from the analysis, since only a few participants in the data collection were in the white zone. As the epidemiological conditions were quite severe, almost no region in Italy had the epidemiological indicators to be classified as a white zone. Thus, it was not possible to quantify spontaneous changes in contact patterns when limited or mild restrictions were implemented. Also, minor changes in the definition of the zones took place during our collection period, and some restrictions were different at the regional level (e.g. school closure was not implemented according to the same criteria for all the regions). However, we expect that these differences will matter to a limited extent since the core of the intervention scheme was well established.

Lastly, we neglected in our analysis the longitudinal nature of the data. While this would allow us to take into account heterogeneity at the individual level (see for example [19]) it would require considering additional effects (e.g. the fatigue associated with repeated surveys) that are not fully understood [15, 20] and we, therefore, leave this for future research. Additionally, in terms of implications for policymakers, our work hasn’t taken into account the assessment of cooperation for example between public and occupational health stakeholders to prevent the effects of COVID- 19 pandemic which has been analyzed more in-depth in other works [21, 22].

## Conclusion

NPIs reduced the number of contacts independently by age group and contact settings. All the interventions considered played an essential role to withstand the pandemic and were able to reduce $R_0$ below one, with stronger measures leading to a greater reduction of $R_0$. As the spread of COVID-19 evolves due to vaccination campaigns and new variants of concerns, continuous monitoring of social contact data can provide invaluable information to quantify the impact of social distancing policies and promptly design effective strategies to reduce the impact of the pandemic.

## Supplementary Information

**Additional file 1.**

## Data Availability

The social contact data analyzed in this survey can be found on Zenodo [23].
