# Supplementary material for: Impact of tiered measures on social contact and mixing patterns of in Italy during the second wave of COVID-19
Source: BMC Public Health. 2023 May 19;23:906. doi: 10.1186/s12889-023-15846-x (PMC10195658; doi:10.1186/s12889-023-15846-x)

Supplementary Informations

March 16, 2023

Sample distribution

In Table 1 we show the distribution of participants for each Italian region across the seven waves of data collection.

Table 1 Distribution of participants per wave and Italian regions.

Region	Wave						
	1	2	3	4	5	6	7
ABRUZZO	18.0 (1.15 %)	15.0 (1.13 %)	14.0 (1.24 %)	13.0 (1.36 %)	11.0 (1.36 %)	11.0 (1.6 %)	7.0 (1.18 %)
BASILICATA	10.0 (0.64 %)	10.0 (0.76 %)	6.0 (0.53 %)	8.0 (0.84 %)	6.0 (0.74 %)	5.0 (0.73 %)	5.0 (0.85 %)
CALABRIA	35.0 (2.25 %)	33.0 (2.49 %)	23.0 (2.04 %)	23.0 (2.41 %)	13.0 (1.6 %)	19.0 (2.76 %)	15.0 (2.54 %)
CAMPANIA	165.0 (10.58 %)	127.0 (9.59 %)	110.0 (9.78 %)	103.0 (10.79 %)	79.0 (9.74 %)	70.0 (10.16 %)	61.0 (10.32 %)
EMILIA-ROMAGNA	121.0 (7.76 %)	105.0 (7.93 %)	96.0 (8.53 %)	81.0 (8.48 %)	65.0 (8.01 %)	53.0 (7.69 %)	42.0 (7.11 %)
F.V GIULIA	35.0 (2.25 %)	32.0 (2.42 %)	25.0 (2.22 %)	21.0 (2.2 %)	21.0 (2.59 %)	20.0 (2.9 %)	11.0 (1.86 %)
LAZIO	150.0 (9.62 %)	128.0 (9.67 %)	118.0 (10.49 %)	83.0 (8.69 %)	72.0 (8.88 %)	76.0 (11.03 %)	63.0 (10.66 %)
LIGURIA	48.0 (3.08 %)	44.0 (3.32 %)	34.0 (3.02 %)	28.0 (2.93 %)	26.0 (3.21 %)	18.0 (2.61 %)	12.0 (2.03 %)
LOMBARDIA	259.0 (16.61 %)	228.0 (17.22 %)	201.0 (17.87 %)	167.0 (17.49 %)	154.0 (18.99 %)	110.0 (15.97 %)	101.0 (17.09 %)
MARCHE	51.0 (3.27 %)	41.0 (3.1 %)	31.0 (2.76 %)	31.0 (3.25 %)	23.0 (2.84 %)	22.0 (3.19 %)	18.0 (3.05 %)
MOLISE	6.0 (0.38 %)	6.0 (0.45 %)	3.0 (0.27 %)	4.0 (0.42 %)	3.0 (0.37 %)	2.0 (0.29 %)	3.0 (0.51 %)
P.A.TRENTO	22.0 (1.41 %)	14.0 (1.06 %)	12.0 (1.07 %)	13.0 (1.36 %)	10.0 (1.23 %)	10.0 (1.45 %)	8.0 (1.35 %)
PIEMONTE	115.0 (7.38 %)	97.0 (7.33 %)	81.0 (7.2 %)	69.0 (7.23 %)	58.0 (7.15 %)	45.0 (6.53 %)	30.0 (5.08 %)
PUGLIA	125.0 (8.02 %)	105.0 (7.93 %)	88.0 (7.82 %)	74.0 (7.75 %)	67.0 (8.26 %)	60.0 (8.71 %)	54.0 (9.14 %)
SARDEGNA	41.0 (2.63 %)	33.0 (2.49 %)	27.0 (2.4 %)	19.0 (1.99 %)	21.0 (2.59 %)	18.0 (2.61 %)	15.0 (2.54 %)
SICILIA	130.0 (8.34 %)	112.0 (8.46 %)	89.0 (7.91 %)	76.0 (7.96 %)	65.0 (8.01 %)	51.0 (7.4 %)	52.0 (8.8 %)
TOSCANA	90.0 (5.77 %)	72.0 (5.44 %)	62.0 (5.51 %)	57.0 (5.97 %)	38.0 (4.69 %)	34.0 (4.93 %)	39.0 (6.6 %)
UMBRIA	18.0 (1.15 %)	15.0 (1.13 %)	13.0 (1.16 %)	11.0 (1.15 %)	12.0 (1.48 %)	7.0 (1.02 %)	7.0 (1.18 %)
VALLE D'AOSTA	1.0 (0.06 %)	1.0 (0.08 %)	1.0 (0.09 %)	1.0 (0.1 %)	1.0 (0.12 %)	2.0 (0.29 %)	_
VENETO	119.0 (7.63 %)	106.0 (8.01 %)	91.0 (8.09 %)	73.0 (7.64 %)	66.0 (8.14 %)	56.0 (8.13 %)	48.0 (8.12 %)

Contact matrices

The detailed contact matrices for the different settings, waves, and zones for adults are shown in Figures (3,1,2) respectively. Due to disaggregation data for children are not sufficient to be included in the matrix, hence the matrices are restricted to the adult population.

R_0 computation and statistical test

In Figure 4 we show an age sensitivity analysis using different susceptibility (a) and infectivity (h) values taken from literature. The formula to compute R_0 is:

$$R_{0_\tau} = R_{0_{ref}} \frac{\rho(diag(\mathbf{a}) M_\tau diag(\mathbf{h}))}{\rho(diag(\mathbf{a}) M_P diag(\mathbf{h}))} \quad (1)$$

In Figure 4-a) we used a = [0.79 0.86 0.8 0.82 0.8] and h = [0.59 0.7 0.76 0.9 0.99] from [1] for Belgium, in figure 4-b) we used a = [0.79 0.86 0.8 0.82 0.8] and h = [0.64 0.67 0.7 0.75 0.84] from [2], while figure 5-c) is the same shown in the

main article where we used homogeneous values equal to 1 (i.e. age independent susceptibility and infectivity). As one can note, the differences between the 3 figures are extremely small because the largest differences in susceptibility and infectivity are between adults and children. However, the latter are not included in our analysis and thus, as one can note, the differences between the 3 figures are extremely small. Furthermore, the couples of vectors a and h used in Figure 4-a) and 4-b) are computed for different countries. For this reason, we kept Figure 4-c) with homogeneous susceptibility and infectivity.

The differences between the matrices in the different zones were measured considering the principal eigenvalue of the matrix as the representative metric. In particular, we computed the t-test and the ks-test to compare the 10000 bootstrap realization of the principal eigenvalues. The distributions of the R_0 samples for each zone is shown in Figure 5. The results show that the matrices are significantly different from one zone to another $p < 0.0001$.

Restrictions

In Figure 6 we show the colors of each region in the periods interested by the waves.

Author details

References

1. N. Franco, P. Coletti, L. Willem, L. Angeli, A. Lajot, S. Abrams, P. Beutels, C. Faes, and N. Hens, "Inferring age-specific differences in susceptibility to and infectiousness upon SARS-CoV-2 infection based on belgian social contact data," *PLOS Computational Biology*, vol. 18, p. e1009965, Mar. 2022.
2. N. G. Davies, P. Klepac, Y. Liu, K. Prem, M. Jit, and R. M. Eggo, "Age-dependent effects in the transmission and control of covid-19 epidemics," *Nature medicine*, vol. 26, no. 8, pp. 1205–1211, 2020.

Figure 1 Contact matrices by waves (1 - 7).

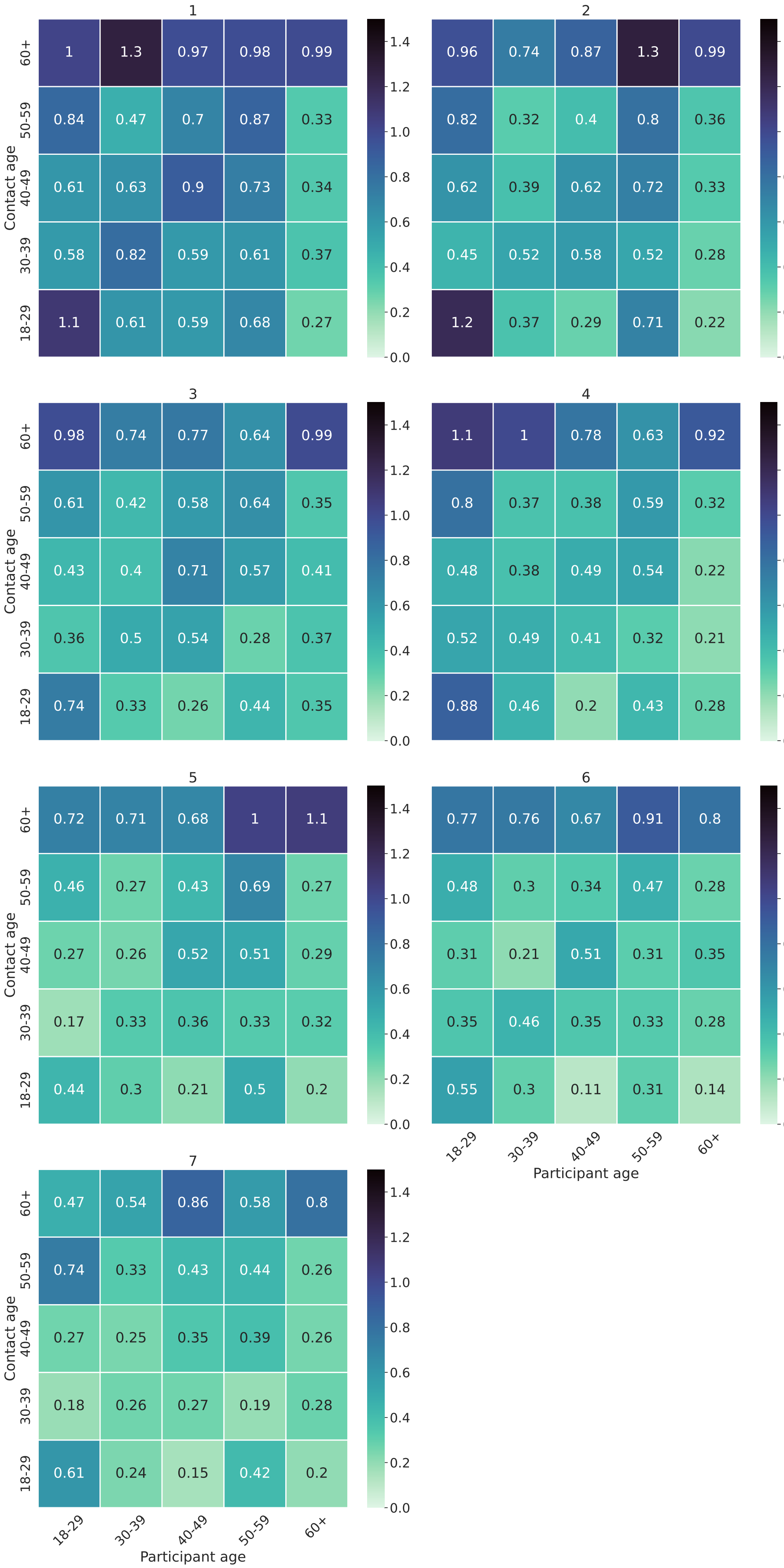

Figure 2 Contact matrices by zone.

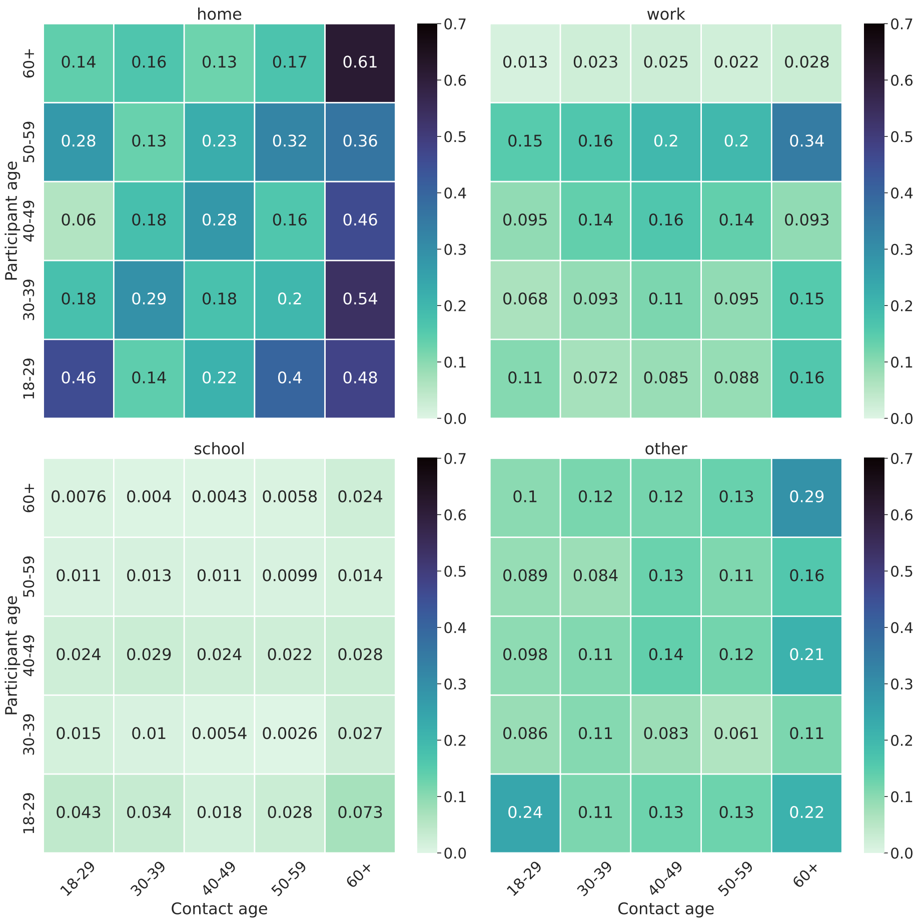

Figure 3 Contact matrices by settings.

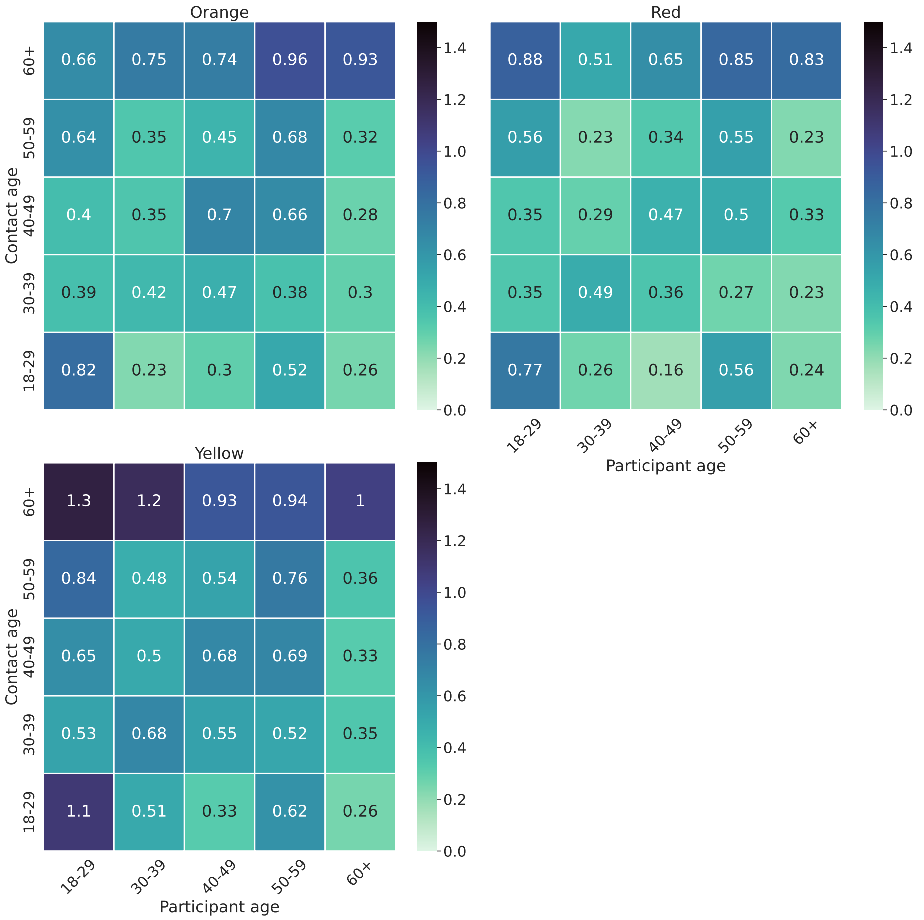

Figure 4 Basic reproduction number for each zone. In Figure a) we used age-dependent susceptibility and infectivity vectors from [1], in b) we used age-dependent susceptibility and infectivity vectors from [2] and in c) we assumed homogeneous values equal to 1 for all age groups.

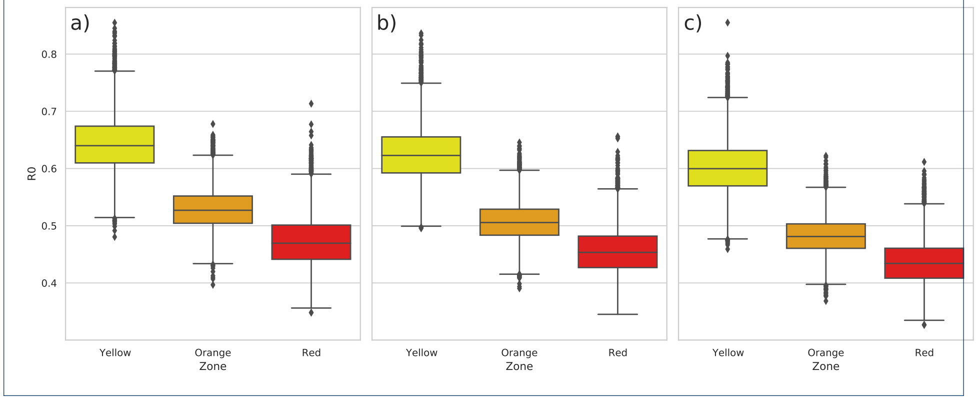

Figure 5 Distribution of eigenvalues for different waves and zones, 10000 bootstrap.

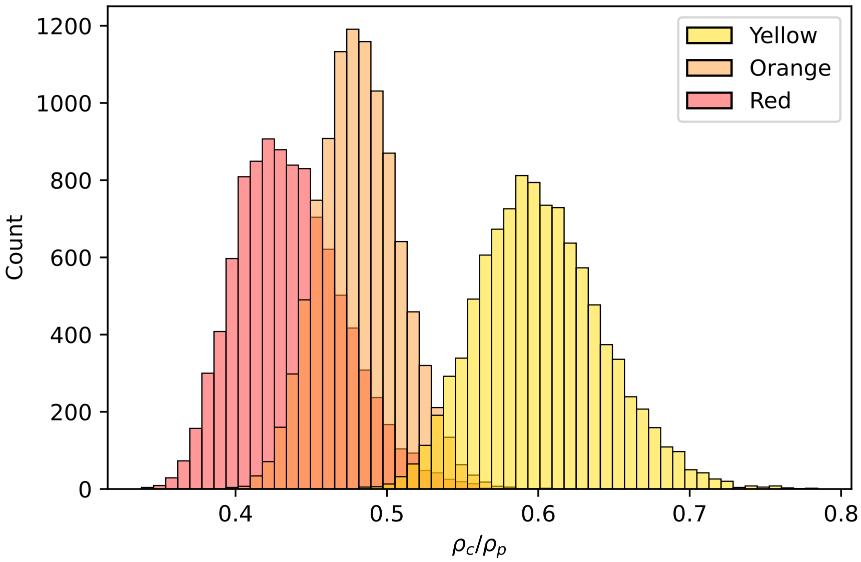

Figure 6 NPIs timeline. Calendar of colors of each region in the period during CoMix waves of data collection. The 7 waves related to adults are on the left, while the 2 waves related to children on the right. The colors match the imposed NPI type for each day of the survey in each region. The color code indicates the NPIs in place in each region, yellow tiles are in the lowest stringency regime, while red tiles are in the highest.

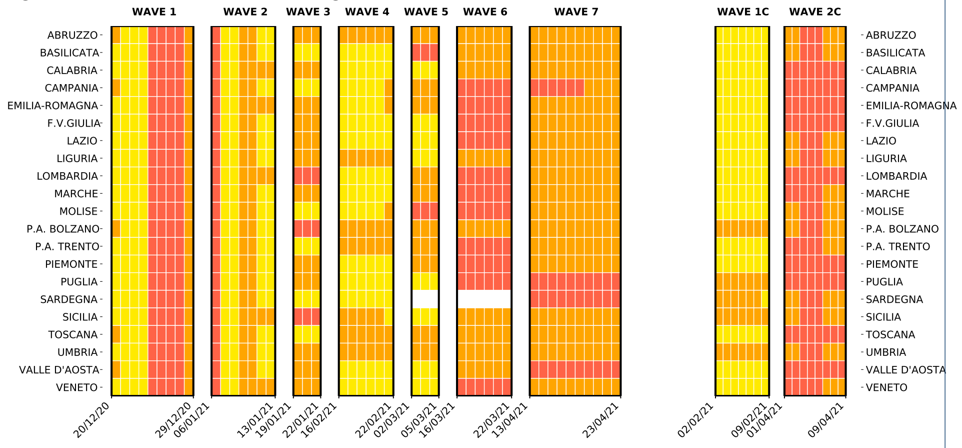

Supplement: Supplementary file 1 — Additional file 1. [file 12889_2023_15846_MOESM1_ESM.pdf]
